# What happened and why? A programme theory-based qualitative evaluation of a healthcare-academia partnership reform in primary care

**DOI:** 10.1186/s12913-019-4665-1

**Published:** 2019-11-01

**Authors:** Håkan Uvhagen, Henna Hasson, Johan Hansson, Mia von Knorring

**Affiliations:** 1 Department of Learning, Informatics, Management and Ethics (LIME), Medical Management Centre (MMC), Karolinska Institutet, 171 77 Stockholm, Sweden; 2 Research and Development Unit for Elderly Persons (FOU nu), Stockholm Region, Sweden; 3 Centre for Epidemiology and Community Medicine, Stockholm Region, Sweden; 4 Unit for Public Health Reporting, Public Health Agency of Sweden, 171 82 Solna, Sweden

**Keywords:** Primary healthcare, Research, Education, Partnership, Implementation, Impact, Programme theory, Evaluation

## Abstract

**Background:**

There is increasing interest in and demands for partnerships between academia and healthcare practices. Few empirical studies have described the influence of such partnerships from a practice perspective. The purpose of this study was to evaluate the impact of a reform launched to increase integration between primary care and academia and to identify potential reasons for why the observed impact occurred in three areas targeted by the reform: research, student education, and continued professional development.

**Methods:**

The study was conducted in Stockholm County, the largest healthcare region in Sweden, at the introduction of a partnership between primary care and academia, including eight coordinating centres and approximately 500 surrounding primary care units. A programme theory-based qualitative approach to evaluation was used, building on document analysis, and in-depth interviews with the centre managers (*n* = 6) and coordinators (*n* = 8) conducted 42–66 months after the initiation of the reform.

**Results:**

The analysis showed that the reform had some impact on all three areas targeted by the reform: research, student education, and continued professional development. The input that contributed most extensively to the impact was the establishment of facilitating roles. Most changes occurred at the coordinating centres and primarily in the area of student education. The effect on student education was primarily due to having prior experience in this area and perceptions of timely benefits of students to care practice.

**Conclusions:**

Partnerships between primary care and academia hold the potential of practice impact. To increase integration between primary care and academia, the components of the integration must be understandable and relevant for primary care practitioners, and importantly, compliant with delivery of primary care.

## Background

Interests in partnerships between academia and healthcare practices to increase quality of care and to contribute to science are increasing [1–7]. Several reports on partnerships between healthcare services and academia that theorize or propose intended impact have been published [2, 3]. However, few empirical studies provide detailed descriptions of such partnerships from a healthcare practice perspective [1, 2, 6, 8]. Many of the empirical studies also focus on research, whereas only a few have addressed education as a key component [9]. Thus the current literature provides an unclear picture of how healthcare practice perceives the impact of initiatives aimed at creating partnerships between practice and academia, and how initiatives including components of student education function in such partnerships.

In this study we evaluate a healthcare-academia partnership initiative called the Academic Primary Healthcare Network (APHN), a regional reform that was introduced in the largest region in Sweden.

### Theoretical perspectives on healthcare-academia partnerships

Partnerships between healthcare and academia could be seen from two alternative paradigms: the transfer and the engagement [10]. These differ regarding roles, engagement and type of collaboration between practice and academia. Within the transfer paradigm, practice, research, and education are regarded as separated activities [10]. Healthcare practice is generally looked upon as a provider of data for researchers, or a clinical setting for students´ training. In this paradigm, knowledge producers (i.e., researchers) are seen as isolated from knowledge users, where knowledge is seen as an achievement less dependent on context [11]. The transfer paradigm is based on the assumption that how knowledge is used is mainly an issue of effective packaging and transfer of knowledge [12]. The paradigm also implies that people will search for knowledge that is produced in universities and other academic settings, find it, and put it into use. In contrast to the transfer paradigm, the engagement paradigm regards practice, research, and education as intertwined activities, linking the generation and use of knowledge closer to healthcare practice [10]. Knowledge is seen as produced in the same context as where it will be applied [12], based on collaborative knowledge generation by academics working alongside other stakeholders with questions framed by those who plan and deliver services together with researchers. The engagement paradigm implies that when healthcare professionals actually participate in research activities or engage in students´ training, they are more likely to use the results and provide positive impact on the quality of care delivered [13].

### Previous research on impact and implementation of partnerships

Regarding impact, the involvement of healthcare organisations and professionals in research and education has been shown to influence healthcare professionals and practices, as well as patients’ health outcomes [4, 14–16]. Partnerships between academia and healthcare services improves patient survival rates [14], organisational performance [16, 17], and job satisfaction among staff members [16, 18–20]. Reported disadvantages include the risk of healthcare productivity losses [19–21] and increased workloads for busy healthcare professionals [19].

Although some studies have shown that partnerships between healthcare practice and academia have a positive impact, such collaborative efforts per se do not directly lead to desirable effects.

Concerning partnership implementation, previous research has identified some key conditions for successful integration of research and education in healthcare practice.

First, it has proven to be advantageous to view partnerships as nonlinear and unpredictable processes, which means working with, rather than trying to simplify or control the embedded complexity of partnerships [2, 22]. This entails continuous local adaptation and modification applying shared measures and constant evaluation, and learning within and across partnership initiatives [2, 12, 23].

Another condition concerns alignment of expectations, because individuals involved in healthcare practice and academia are likely to promote collaboration based on their potentially different experiences and interests [13, 24]. Hence, important conditions include leadership actions for evolvement of relationships and trust within the collaboration [2, 12, 22, 25], as well as continuous clarification and alignment of goals and expectations [2, 12]. Still, despite the need for mutual respect and understanding of expectations, it seems unlikely that stakeholders become engaged in collaborative processes without being clear over “what’s in it for me” [2].

How partnerships are performed is another condition for successful integration. Rather than separating the production and the use of research in healthcare practice, a collaborative approach has been suggested to facilitate the pathway leading from engagement in research and education to improved healthcare performance [4, 7, 11]. Involving both research producers and users at an early stage can generate practice-relevant research questions and, eventually, also knowledge that is transferable, relevant, and usable for healthcare professionals and patients [12, 13, 19, 26, 27]. Such an approach implies complementary roles and contributions for both researchers and healthcare professionals throughout the entire research and implementation process [6].

To achieve partnerships in healthcare organisations, resources are needed not only for routine service delivery, but also to facilitate academia-care integration [2, 12, 15, 28, 29]. Besides funding, the resources in this context include access to methods and tools, the right expertise and skills, adequate time for establishment of partnerships, and also support for partnership development in the longer term [12]. One example of a facilitation arrangement involves the use of intermediary bridging, brokering, and boundary-spanning researcher roles [1, 2, 30–33]. In contrast to strict academic research positions, which entail working exclusively within a university institution, the activities in boundary-spanning roles are performed directly in a healthcare setting. In general, these facilitator roles are intended to make the partnership process easier for professionals in healthcare practice by helping them change their way of thinking and working [2, 15, 30, 31, 34].

In summary, previous research has found both positive and negative impact from and conditions for partnerships between healthcare and academia. However, only a few of those studies have investigated the actual impact of such partnerships from a practice perspective or identified the mechanisms that need to be established to obtain an intended impact. Partnerships do turn out as intended in some cases, but not always, although we still do not know why [4, 35].

### Study aim

The aim of the study is to evaluate the impact of a reform launched to increase integration between primary care and academia and to identify potential reasons for why the observed impact occurred in three areas targeted by the reform: research, student education, and continued professional development (CPD).

The term impact here refers to both outputs (i.e., direct products of reform activities) and outcomes (i.e., actual benefits – or potential disadvantages – resulting from the reform activities and outputs) [36].

## Methods

### Design

We used a programme theory-based qualitative approach to evaluation to evaluate the impact and identify why the impact occurred [37].

### Setting

The study was performed in Stockholm County, Sweden. Primary care in Stockholm includes community health centres, preventive care, rehabilitation, home care services, child health services, and maternal health services [38], and it comprises approximately 500 care units representing both public and private care providers.

The Academic Primary Healthcare Network (APHN) reform was introduced in Stockholm County in 2011 by policymakers and the senior management team in the primary care organisation. The reform was funded by the local primary care organisation and governed in collaboration with the local medical universities. The reform aimed to achieve integration between primary care and academia by increasing and stimulating research closely connected with primary care, and improving coordination and structures necessary for both clinical training of students and CPD of staff. Two main issues to be addressed by the reform were to improve chances to recruit and keep staff, and to improve the quality of care [39, 40].

Four coordinating centres were launched in 2011 to oversee organisation of networks of primary care units, and four additional coordinating centres were started in 2014. Each of the eight centres covered approximately 60 primary care units located within a specific geographical area. The managers of the coordinating centres were responsible for the reform implementation, and, throughout this process, the eight centres continued with their regular healthcare assignments.

One main input was the appointment of a part-time coordinator at each coordinating centre. It was stipulated that a coordinator was to have a doctoral degree and be affiliated with a regional medical university but employed in primary care. The task of the coordinator was to promote, coordinate, and improve education and research in collaboration with the universities and primary care staff.

### Data collection and analysis

To illustrate the theory behind the reform and its intended outcomes we collected steering documents used at the initiation of the reform in 2011. Based on document analysis of that data [41], a preliminary logic model was then constructed [42]. The preliminary logic model was used to illustrate the underlying idea of how the reform was expected to work (i.e., the programme theory) [42] by displaying a flow chart with the expected steps from reform inputs and activities to intended outputs and outcomes.

To ensure that we had correctly identified the theory of the programme (in this case the reform), we then compared our findings in the preliminary logic models with data from previous interviews regarding the intentions of the reform and its stated objectives. These interviews had been conducted with coordinating managers (*n* = 8) and coordinators (*n* = 4) during early phases of the reform (i.e., 2013–2014). The comparison with the interview statements confirmed the preliminary logic model (see Additional file 1) and suggested no changes to the preliminary logic model.

In the next step, we collected new data to identify how the expected impact of the reform was realized in practice. The preliminary logic model was used to inform the development of an interview guide, which was constructed to explore how the respondents perceived the outputs and outcomes of the initiative, and what they thought contributed to these changes (the interview guide is presented in Additional file 2). Interviews were conducted in May and June 2017 with managers and coordinators at the coordinating centres, approximately 42–66 months after initiation of the reform. All 16 managers and coordinators at the eight centres received an email that described the aim of the interviews and invited participation. Fourteen accepted participation. Eight of those had also taken part in the previous interviews conducted in 2013–2014, whereas the other six were new staff members and had not participated in the earlier interviews. The respondents consisted of nine women and five men. Six were managers and eight were coordinators. The time they had participated in the APHN initiative ranged from approximately 1 year to five and a half years. Thirteen interviews were conducted face-to face, and one interview was performed by telephone for practical reasons. The first author carried out all but two of the interviews, and those two were conducted by the last author.

The interviews were transcribed verbatim. In the first step, data on how the respondents perceived how the reform was realized in practice was identified using a thematic analysis approach [43]. The findings were then framed within the logic model, and compared to the expected impact as described in the preliminary logic model [42]. For statements that described impacts that were only partly achieved in practice, the specified impacts were denoted as being realized to only a limited degree. In the next step of the analysis, data reflecting the respondents’ views on what had contributed to the impact were coded and categorised thematically [43]. This step of the analysis addressed both the respondents’ descriptions of the reform impact and the specific linkages between the elements in the logic model. The analysis helped to better understand the reasons and mechanisms that linked inputs, activities, outputs to outcomes.

In the following section, we illustrate the findings by using quotes, all of which have been read and validated by the respondents. Each quote has been assigned a letter and a number designating the specific respondent (managers M1–M6; coordinators C1–C8). In the quotes, the symbol / … / indicates an omission, and [] indicates an addition to the transcript excerpts; these changes have not altered the meaning of the quotes and were made solely to enhance readability or secure the anonymity of the respondents. All quotes presented here have been translated from Swedish.

## Results

In the following, we first present a logic model illustrating how the reform was realized in practice (see Table 1). Thereafter we give a more detailed analysis of the overall impact of the reform. Finally, we outline the identified reasons for why that impact occurred.

**Table 1:** The logic model illustrating how the reform was realized in practice

Inputs	Activities	Outputs	Outcomes
*Definition: Resources provided to achieve the reform activities but not previously available for this purpose*	*Definition: Activities, not done before, to achieve reform outputs and outcomes*	*Definition: Direct products of the reform’s activities that otherwise would not have happened*	*Definition: Actual benefits – or potential disadvantages – resulting from the reform activities and outputs*
*) Steering document clarifying the mandates *) The project group, the steering committee, and the reference group not mentioned as influential *) Research and educational competencies not present to the extent that was intended Resources for coordinators and clinical lecturers Start-up funding (e.g., for targeted ventures and learning environments) ***) Lack of prerequisites (e.g., time, competence, and facilities) to be able to combine engagement in research, education, and professional development with delivery of care services	Establishment of eight coordinating centres Establishment of eight networks Extended mandate to the managers of the coordinating centres Establishment of eight coordinators for the centres Establishment of coordination of clinical lecturers ***) Lack of a systems perspective parallel to local APHN development	Students’ clinical training: Improved coordination and structure, improved competencies (e.g., increased number of trained supervisors), more students, improved learning environments (e.g., educational settings, establishment of practices managed by students) *) Attempts were made to increase students’ inter professional training, but there were difficulties in establishing continued systematic approaches Continuous professional development (CPD): More opportunities and activities Research: *) Research projects with connection to primary care were initiated but difficult to run in practice, although some practitioners did become engaged in the research Other: *) Networks were established but had few activities; some collaboration was established with actors outside the primary care organisation (e.g., social services); **) increased collaboration with actors outside the primary care organisation (e.g., research collaborations, introduction of technical developments), and further specification of the local profiles at the centres	Increased dialogue about improvement of care quality and the use of evidence-based interventions, although in its early stages More positive attitudes towards students and research in primary care **) Increased workload (e.g., more tasks and time-consuming activities) Improved job satisfaction and chances to recruit and keep staff (e.g., job variation, confidence in supervising roles, increased personal development and competence)

Regular text indicates the changes that were described in the preliminary logic model and also realized in practice; text noted *) was described in the preliminary logic model but only to a limited degree realized in practice; text noted **) was not described in the preliminary logic model but was realized in practice; text noted ***) was not described in the preliminary logic model and was not put in place but was highly missed by the respondents

### Impact of the reform

Overall, the analysis showed that the reform had some impact on all three targeted areas: research, student education, and CPD. However, there were also some other impact, not specifically related to the areas targeted by the reform. Below, the identified impact will be described.

#### Increased focus on students

An intended output of the reform was to improve coordination and structure of students’ clinical training and increase the competencies of primary healthcare staff for providing such training. The analysis showed that this was achieved. The interviewees reported an improvement of clinical training structures and pedagogic competencies and that this had helped to clarify the roles of the staff members in student training. They also reported that the improvements had led to an increased number of students participating in training activities.

Instead of it being a matter of “oh, do we have a student here today, oh, that’s right, you’re the one who’s responsible for this one”, it sort of implies an organisation in which it’s very clear who is to introduce the student, and what has to be done to ensure that the introduction is meaningful and helps the student take part in activities as quickly as possible. (C1).

The analysis also identified improvement in the learning environments for students. One example was the introduction of care units that were managed by students (rather than professionals), which enhanced student satisfaction and facilitated structured collaborations and dialogues between primary healthcare and academia.

The interviewees also described attempts to increase inter professional training for students, however, this seemed difficult to achieve in practice.

That [inter professional training] is a popular ambition, but how realistic and important it is, I haven’t really [considered] yet. I mean it’s difficult [to accomplish], and I think that it’s more difficult to achieve that goal in primary care than maybe in a hospital clinic, because you know, there isn’t enough time to make it work, and you have to manage your duties all the time to keep things running. (C3).

Taken together, the stronger focus on education and improved coordination and structures for clinical training gave an enhancement of practice quality. Students brought new perspectives to care and asked questions that countered traditional ideas and ways of working. This led to changes in attitudes and understanding of students and research as parts of primary care. Some reported that the view of regarding research and students as “disturbing” activities were changing.

We are taking part in a cultural change in which students are regarded as valuable, the more we can manage the better / … /. So that teaching and the students represent an important, agreeable, and prioritised aspect rather than a burden. (C5).

#### Facilitation of CPD activities

Another intended impact of the reform concerned providing more opportunities for CPD. The analysis showed that this was achieved. For example, the coordinators facilitated care professionals getting together more often. For the staff at a primary care facility, this helped reduce the amount of time they had to spend on travelling to attend meetings.

[The coordinators and the clinical lectures] have pointed out the significance of further professional training, because it is important for all staff members, and I think that this has become more apparent [since the APHN reform]. (M1).

#### Limited focus on research

An additional intended impact of the reform was that it would lead to more research closely connected with primary care. This objective was achieved to some extent. Both extensions of ongoing research and some new research projects associated with primary care were initiated, which added to more continuous research activities. There were also examples of practitioners who started PhD training, due to the reform.

The research was connected to primary care in different way ranging from early involvement of care staff in the design of the study, to staff functioning mainly as data providers. However, research activities seemed difficult to implement, and, in contrast to education, these activities were described as being few and in early stages.

Research has been performed and initiated, although not very comprehensively or the way we wanted it. But I still feel like it’s a start, a start period. (C4).

#### Other general impact

The analysis also showed some other impact, not specifically related to research, student education, and CPD. Although the intention of the reform was to establish networks of care units to spread and facilitate the research and learning potential of the reform, these networks seemed only to a limited degree realized in practice.

Well, like most of the others, [I think that] the networks have been something very difficult / … /. So that the vast majority of the primary care centres never participate in the network meetings. (C1).

However, in some cases increased collaboration was established with other actors outside the primary care organisation (e.g., social services and other healthcare sectors). This was not described as an intended outcome, but was realized in practice.

Another unexpected output was that the established centres further specified their local profiles based on unique strengths and local interests. This impact seemed to build on what was already familiar at the centres.

Further, the analysis showed that the reform, although at early stages, seemed to result in increased dialogue about quality improvement and efforts based on evidence-based interventions and scientific knowledge.

I feel that [the reform] has awakened an interest that already existed somewhere in the staff – if you put it that way, it was so well hidden. Just needed a spark to somehow get things started and move forward. (M3).

One additional outcome that was not intended in the reform was an increased workload. All of the interviewees mentioned, in one way or another, that it was stressful and difficult to manage the delivery of primary healthcare and at the same time engage in research, education, and professional development, all in the context of a busy practice with staff shortages.

In the beginning, it was just demanding for the care centre. Considering resources, you have to get a hold of substitutes, it gets chaotic, a heavy situation in the clinic. (M2).

On the other hand, participation in research and education and a clearer role as a student supervisor was emphasized as increasing job satisfaction. Staff development, more confidence regarding participation in research and clinical training, and more variation in work tasks were considered to contribute to a more attractive workplace and improved chances of recruiting and keeping staff.

Taken together, the analysis shows that the reform had some impact on all targeted areas: more research with a connection to primary care was conducted; improved coordination and structures for clinical training of students; more opportunities for CPD of staff. However, most of these changes occurred in the area of student education and mainly at the coordinating centres, and based on their already existing centre profiles. This indicates that the reform mainly had impact on the local level, rather than on system levels.

### Reasons for why that impact occurred

Several reason for why the impact of the reform occurred could be identified. Below, these reasons will be described.

#### Reasons for primarily focusing on education

The analysis showed that the reform so far have had most impact in the area of coordination and structures for clinical training of students (rather than research or CPD). The reason for this seemed based on the fact that education was the area that was most familiar for the primary care centres. Education was intuitively comprehensive, and improvement in this area required less resources to achieve. Thus, starting with education seemed to be the easiest strategy for primary care.

I also think that one of the reasons is that it’s been relatively simple / … /, we’ve had three assignments, it’s about research, professional development, and students. And there have already been students here [in primary care], so in a way they are already on location, / … /, the other two parts are a little more, require a bit more than just starting off with this type of activity. (C6).

The focus on education also addressed the burning issues of staff recruitment in primary care (as students were considered important and potential future staff members). Thus, developing education as such was considered important but also relevant to the other reform areas.

#### Reasons for having limited research

In contrast to education, research was described as “something new” and therefore expected to be resource-demanding and time-consuming with limited return of investments for primary care and the service users (i.e., the benefits for care services were not clear).

You could say that there’s kind of a fear among us [in primary care] / … / that this thing with research is something that you do in a laboratory, far away from patients. And that it’s something unfamiliar and not rooted in reality. (C7).

Although it was clearly stipulated that the coordinators at the participating centres were to have a background in research (i.e., a PhD degree), it seems that employing coordinators with research backgrounds was not sufficient to have a pronounced impact on primary care, a setting where research is regarded as somewhat unfamiliar. Furthermore, the eight coordinators described themselves as isolated in their units, which led to difficulties for them to start a change of the culture on their own.

The interviewees also reported a lack of time for practitioners to engage in research and concurrently provide patient care, without jeopardising healthcare availability, patient safety, and working conditions.

… it’s also a burden on the ordinary regular care services, this is an extra task. It’s heavy, it isn’t funded as it should be, and many family doctors say no “I don’t have the strength”. (M2).

Although research was conducted to a limited degree, the importance of using co-creative research approaches that started from a practice perspectives (i.e., practical needs and problems) was emphasised. This approach resulted in research that was perceived as more practice relevant.

Again, what is very satisfying is viewing this process from a “bottom-to-top” perspective, because it’s in the clinical interaction with patients that we find both what is actually needed and the questions of interest, and quite often also suggestions for solutions. (C5).

### Reasons for specification of the local profiles of the centres

One unexpected impact of the reform was further specification of the local profiles of the centres. The interviewees described the reform as being vaguely formulated (i.e., giving unspecific guidance on what to achieve), which gave the centres freedom and flexibility to define the content of the initiative to fit the local context. This also implied that the centres could define their own priorities regarding the different goals and activities in the reform.

The APHN reform had both positive and negative sides, the negative being that there was a lack of structure, and the positive that it was possible to shape things. (C4).

To some extent, the above-mentioned prioritising started in focus areas in which the centres were already established. The interests of a centre and its coordinator also often served as the basis for choosing what area to focus on. Thus an area that was already strong at a centre was further strengthened by the reform.

In a longer perspective, the local profiles entailed limitations: the further the local profiles were developed, the more difficult it became to agree on shared efforts and establish extensive collaboration between the centres. This seemed based on a lack of an overall steering towards a common goal.

… and then of course / … / there can be different goals, and to some extent “all flowers should be allowed to bloom”, but in a way I think that there still has to be a common core. (C8).

#### Reasons for local rather than system level changes

The impact of the reform was most extensive at the eight centres where the coordinators were stationed, and there were fewer changes at the other units. This implies that the reform that was aimed at achieving system-level changes had the greatest impact on some specific parts of the system rather than on the system as a whole. The analysis showed that one reason for such an effect was a limitation in the form of a critical mass of persons with academic experience at each centre. It proved difficult to achieve the logic that one individual (i.e., a part-time coordinator) can reach out from one unit and change several (i.e., approximately 60) other units. Another reason was a lack of incentives for the units in the networks to collaborate. The reform assignment was given to the coordinating centres, whereas no incentive was described on a system level.

It’s very difficult for a coordinator / … /. I think that there might be some overconfidence in how much one person can achieve. (C1).

### The importance of facilitating roles to achieve impact

However, despite the difficulties described above, the aspect that seemed to contribute most extensively to the overall impact of the reform was the establishment of the facilitating roles (i.e., the eight coordinators for the centres and appointed clinical lecturers). Inasmuch as primary healthcare practice alone had limited time and competence to engage in education, research, and continuous professional development, these activities made it possible to initiate the reform by taking part in the integration and also strengthened the skills and knowledge of the actors involved so that they could get started.

The services in primary care have clearly dictated financial resources, and therefore other individuals are needed who can work on these things [education, research, professional development], and I think that this has been shown very clearly. (M1).

The coordinators promoted the integration by building relationships with primary healthcare practice, and this was accomplished through being close to practice (i.e., not only physically “located in” a centre but present as an embedded “part of” primary healthcare).

You have to know the individuals to kind of cross the threshold. You can’t just come from somewhere else and sort of say let’s get started, and then just leave. Then you’ll never gain anyone’s confidence. (C6).

By having experience of both academia and healthcare practice, the coordinators gained credibility and also embodied the integration. The interactions between the coordinators and the practitioners made it possible to discuss education and research in a way that made sense to the practitioners. The coordinators’ academic training was also an advantage when applying for research funding and conducting and supervising research. The interviewees indicated that such activities would not have been possible without presence of the coordinators, implying that there was not enough time or skills to be achieved by primary care alone.

## Discussion

This study shows that the reform had some impact on all the areas that was initially targeted in the reform. However, when comparing what happened in practice with what was articulated in the programme theory, it was clear that while some inputs and activities were put in place others were missing and some impact happened as intended whereas other was unexpected or only happened to a limited degree.

We found that the most evident impact was in the area of student education. A possible explanation for this could be that it was the easiest area to start with, could be managed with the regular delivery of care, and required limited resources (compared with research or CPD of staff). Investing in student training was also perceived to have more a timely impact on improvement of care quality and other burning issues in primary care, such as recruitment of new staff. As shown in previous studies, dealing with students in a structured manner and showing them an attractive workplace might enable recruitment of future colleagues [44, 45]. The significance of prior experience for future choice of improvement areas has been described in previous studies of similar types of partnerships [2, 35]. Furthermore, obtaining rapid results in the form of improvement has seems to be a central issue in theories of motivation. For instance, Michie et al. [46] explained how experience of positive outcomes of an intervention affect behavioural direction and individuals’ motivation to continue changing their behaviour.

The relatively limited impact on research found in this case, could be explained by the same mechanism mentioned above. Compared to education, research was perceived as being less familiar and requiring more resources and expertise, and was often described as having little value for anyone but the researchers themselves (i.e., the benefits for care were ambiguous). Perceptions of research as an activity that has its own agenda, separate from care practice, has been described in previous research on healthcare-academia partnerships [19]. In sum, it seems as student education is a more easily part of the traditional primary care role than research.

Our findings also showed that the coordinating centres had specific profiles, particularly in relation to the research that was performed. These profiles emerged partly because some of the centres were already focused on certain areas, which illustrates that partnerships are influenced by history and by previously established partnerships [2, 47]. Other centres in our study chose to develop a specific profile after introduction of the reform based on the coordinators’ interests. The specification was possible, because the reform was flexible and gave only general guidance regarding what was to be achieved. In addition, the overall steering of the reform implementation towards the common goal was limited and in some instances was perceived as absent. This gave the centres freedom to interpret the reform to fit to their local context. The flexibility of the reform offered the advantage of getting the involved centres on track. However, this became problematic in that the local profiles limited collaboration and sharing of activities between the centres. This corresponds with previous research showing that it is difficult to change a path once it is set [12] and also highlights the importance of having a common overall goal to bear in mind when developing local profiles.

The original APHN programme theory outlined several system-level changes. However, the impact occurred mainly on some specific parts of the system, i.e. at the coordinating centres. Given this, one key question for policymakers is how to design reform strategies that can enable changes on a system level. Our study provides some answers by showing that solid planning and overall management are important in this context. Other investigations have outlined the significance of suitable contextual conditions and sufficient resources, as well as ensuring involvement of stakeholders and the use of continuous system feedback to make intended system changes happen [48, 49].

Our findings show that the input and activity that seemed to contribute most extensively to the overall impact of the reform was the establishment of the facilitating roles. The importance of facilitating arrangements have been extensively reported in previous research [1, 2, 30–33]. In a study of partnership implementation in primary care, Malone et al. [2] concluded that investments in such roles resulted in facilitation and direct partnership impact.

Previous research on healthcare-academia partnerships has been conducted, explicitly or implicitly, in relation to one of two alternative paradigms, the transfer and the engagement paradigm [10]. Research conducted within the engagement paradigm builds on the assumption that mutual engagement and collaboration on equal terms between healthcare practice and academia results in more relevant research questions and the creation of knowledge that is more readily transferable, relevant, and usable for practice [2]. In this study, we can identify impact in a number of areas, despite the fact that limited research activities were actually initiated. This finding implies that impact of healthcare-academia partnerships initiatives occurs early in the implementation process by primary care being an active part in the partnership process. This strengthens the arguments for partnership initiatives that are grounded in the engagement paradigm where the emphasis is put on co-production and early engagement between academia and primary care practice.

Still, to build collaboration between healthcare practice and academia on the engagement paradigm cannot be regarded as a quick fix or easily done [12, 13, 50]. As reported here, such an approach implies a major shift in traditional roles that demands the engagement, time, and persistence of all stakeholders [6, 13, 28, 51]. This situation seems to put healthcare practice in a tradeoff situation between an active and resource demanding role that is likely to interfere with care practice (following the engagement paradigm) with the possibility of beneficial impact already by partnership engagement. Another option for care practice is a less active and less resource demanding role that is likely to interfere less with care practice (following the transfer paradigm). However, previous research increasingly point out that the idea of knowledge production as a linear process that is being achieved separated from the knowledge users, packaged, transferred, picked up, and then finally used is an oversimplified view of a complex process [10, 12, 22, 52].

In some cases, research may require a closer connection between academia and practice, but that does not necessarily mean that a collaborative approach must be applied [2]. Future research is still needed to fully understand what types of partnership approaches are the most productive and in what contexts they can be applicable [6, 10, 53].

### Methodological considerations

In this study we used the perceptions of the managers and coordinators involved to evaluate the impact of a reform and to analyse why the observed impact occurred. An even broader perspective could have been achieved if staff members at both the coordinating centres and the primary care units in the networks had been included. Additional data from other data sources, e.g., students´ evaluations of their clinical training and data on care quality from the national primary care quality register, might also have strengthened the study results [42]. An advantage of the study, however, is that all coordinators and almost all managers at the coordinating centres in the reform were interviewed [54], allowing richness in our findings.

An additional strength is that all quotes used in the present study were read by the individuals who made the statements which increases validity. Furthermore, all the authors read the transcripts and helped identify and explain impacts of the reform, which further strengthens the validity of the results.

The programme theory-based approach to evaluation and the logic model that was used as a framework for data gathering and analysis, helped identify the relevant activities, outputs, outcomes, and linkages to be included in the evaluation, and also provided a structure for the analysis [42, 55]. A strength of the study is that the logic model was discussed by all the authors to assure that all relevant aspects were covered. Another strength is that the preliminary logic model that was based on the document analysis, was tested against statements from interviews performed in the initial phase of the reform which increased the validity of the model. The model might have been further refined through additional interviews concerning the model with those involved in the reform.

Measuring how an intervention has contributed to identified changes is challenging and interventions are seldom, if ever, as linear as depicted in a logic models. Still, using a logic model as starting point and using the perceptions on these involved in an implementation can help identify the conditions that are critical for achieving desired outcomes, unintended impacts, and provide explanations for what has actually happened [37].

It is noticeable that the reported impact of the reform are service based. Further studies using additional data sources that enable quantifiable measures to explore, for example, impact on patient outcomes are needed.

The current findings should be interpreted in the context of the implemented reform. Even though conducted in a Swedish context, the findings should have relevance also for other context where partnerships between primary healthcare and academia is initiated.

## Conclusions

This study shows that partnerships between primary care and academia hold the potential to have an impact on healthcare practices. However, impact does not seem to happen by itself. To succeed with healthcare-academia partnerships, primary care needs help from facilitating roles located in care practice, but it is not enough. To be able for healthcare practice to engage in partnerships and to increase integration between primary care and academia, the components of the integration must be understandable and relevant for primary care practitioners, and importantly, compliant with delivery of primary care.

## Supplementary information

**Additional file 1:** The preliminary logic model illustrating the idea behind how the Academic Primary Healthcare Networks were expected to work (i.e., the programme theory).

**Additional file 2:** Interview guide.

## Data Availability

The dataset generated and analysed during the current study are not publicly available since individual privacy could be compromised but are available from the corresponding author on reasonable request.

## Abbreviations

APHN: Academic Primary Healthcare Network
CDP: Continued Professional Development
