# Supplementary material for: What happened and why? A programme theory-based qualitative evaluation of a healthcare-academia partnership reform in primary care
Source: BMC Health Serv Res. 2019 Nov 1;19:785. doi: 10.1186/s12913-019-4665-1 (PMC6825344; doi:10.1186/s12913-019-4665-1)
Supplement: Supplementary file 1 — Additional file 1: The preliminary logic model illustrating the idea behind how the Academic Primary Healthcare Networks were expected to work (i.e., the programme theory). [file 12913_2019_4665_MOESM1_ESM.docx]

**Additional file 1:** The preliminary logic model illustrating the idea behind how the Academic Primary Healthcare Networks were expected to work (i.e., the programme theory)

| **Inputs** | **Activities** | **Outputs** | **Outcomes** |
| --- | --- | --- | --- |
| Mandate described in a steering document  Establishment of a steering committee, a project group, and a reference group  Research and educational competencies from academia  Funding for coordinators and clinical lecture resources  Funding for initial start-up | Establishment of eight coordinating centres  Establishment of eight networks  Extended mandate to the managers of the coordinating centres  Establishment of eight coordinators for the centres  Establishment and coordination of clinical lectures | **Students’ clinical training** Improved coordination, structures, and learning environments for students’ clinical training, and improved competencies in clinical training  More students in clinical training  Increased inter professional training for students  **Continuous professional development** More continuous professional development opportunities and activities  **Research** More research projects with connection to primary care  **Other** Establishment of networks of primary care units  Collaboration with actors outside primary care organisations | Increased dialogue about improvement of the quality of care and the use of evidence-based interventions  More positive attitudes towards students and research in primary care  Improved job satisfaction  Better chances of recruiting and keeping staff |
