# Supplementary material for: What happened and why? A programme theory-based qualitative evaluation of a healthcare-academia partnership reform in primary care
Source: BMC Health Serv Res. 2019 Nov 1;19:785. doi: 10.1186/s12913-019-4665-1 (PMC6825344; doi:10.1186/s12913-019-4665-1)
Supplement: Supplementary file 2 — Additional file 2: Interview guide. [file 12913_2019_4665_MOESM2_ESM.docx]

**Additional File 2: Interview guide**

***Introductory Questions***

You have been appointed head/coordinator of an Academic Primary Healthcare Network (APHN), and we start from that point.

- With an open mind, what would you say was achieved by introduction of the APHN that otherwise would not have accomplished?

In your opinion, why was that achieved?

***Main questions***

The APHN reform includes integration of research, clinical training of students, and continued professional development of staff.

- Considering the research part of the reform, can you describe what has been achieved by introduction of the APHN that would otherwise not have been accomplished?

In your opinion, why was that achieved?

- The APHN assignment states that more research projects should be performed in connection with primary care.

How do you feel about that?

In your opinion, why was that achieved?

- Considering the clinical training part of the reform, can you describe what has been achieved by introduction of the APHN that otherwise would not have been accomplished?

In your opinion, why was that achieved?

- The APHN assignment states that inter professional training for students, the number of students in clinical training, and competencies regarding clinical training should be increased.

How do you feel about that?

In your opinion, why was that achieved?

- Considering the part of the reform concerning continued professional development, can you describe what has been achieved by introduction of the APHN that otherwise would not have been accomplished?

In your opinion, why was that achieved?

- Establishment of networks of primary care units is described in the APHN assignment.

How do you feel about that?

In your opinion, why was that achieved?
